# The translational regulation of maternal mRNAs in time and space

**DOI:** 10.1002/1873-3468.13183

**Published:** 2018-07-12

**Authors:** Cecilia Lanny Winata, Vladimir Korzh

**Affiliations:** ^1^ International Institute of Molecular and Cell Biology in Warsaw Poland; ^2^ Max‐Planck Institute for Heart and Lung Research Bad Nauheim Germany

**Keywords:** cytoplasmic polyadenylation, maternal mRNA, maternal to zygotic transition, translational regulation

## Abstract

Since their discovery, the study of maternal mRNAs has led to the identification of mechanisms underlying their spatiotemporal regulation within the context of oogenesis and early embryogenesis. Following synthesis in the oocyte, maternal mRNAs are translationally silenced and sequestered into storage in cytoplasmic granules. At the same time, their unique distribution patterns throughout the oocyte and embryo are tightly controlled and connected to their functions in downstream embryonic processes. At certain points in oogenesis and early embryogenesis, maternal mRNAs are translationally activated to perform their functions in a timely manner. The cytoplasmic polyadenylation machinery is responsible for the translational activation of maternal mRNAs, and its role in initiating the maternal to zygotic transition events has recently come to light. Here, we summarize the current knowledge on maternal mRNA regulation, with particular focus on cytoplasmic polyadenylation as a mechanism for translational regulation.

## Abbreviations

**CPE**, cytoplasmic polyadenylation element

**CPEB**, CPE‐binding protein

**GV**, germinal vesicle

**MBT**, mid‐blastula transition

**MPF**, maturation promoting factor

**MZT**, maternal‐to‐zygotic transition

**PAS**, polyadenylation site

**SCA**, subcortical aggregates

**snRNA**, small nuclear RNA

**Figure 1:**
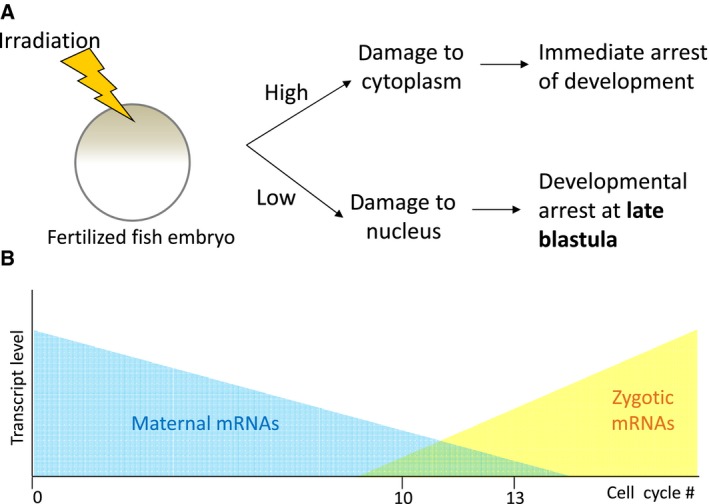
Maternal mRNAs control development up to the point when zygotic genome is activated during the MZT occurring at late blastula in fishes and amphibians. (A) Neyfakh's experiment with irradiated loach embryos show different phenotypes depending on the doses of radiation which affects either cytoplasm or nucleus. The delayed developmental arrest resulting upon nuclear damage gave rise to the understanding of the morphogenetic function of the nuclei, which is responsible for development from late blastula onward. (B) Current understanding at the molecular level established that maternal mRNAs present at high levels prior to MZT drives development up to this point, before it is taken over by zygotic genes expressed from here onward.

In the early 1950s, Alexander Neyfakh observed that different doses of X‐ray irradiation cause different effects in the fertilized eggs of the loach (*Misgurnus fossilis*) (Fig. 1A). A lower dose caused damage to the nucleus and triggered the arrest of development at late blastula stage. A higher dose of irradiation caused damage to the cytoplasm followed by an almost immediate developmental arrest 1. Neyfakh's observation became the basis for the idea of a separate genetic function of the nucleus and cytoplasm, where the former (defined as the ‘morphogenetic function’) required only starting from late blastula, and the latter being indispensable from the very first steps of development. This idea, which had been validated by X‐ray irradiation studies in fish, amphibians, echinoderms, worms, and molluscs 2, was in line with the observations that embryos resulting from interspecies hybrids of fishes, amphibians, and echinoderms stop developing at approximately the same stage of late blastula 3, 4, presumably due to the discrepancy between their cytoplasmic and nuclear genetic programs.

Depending on the size of an egg and the amount of cytoplasm deposited into an egg by the mother in the species studied, fertilization is followed by a period of transcriptional quiescence of varying length when the genome of the embryo which is contained in the nucleus is not yet expressed. During this period of transcriptional silence, development is driven by cytoplasmic factors which largely consist of maternally deposited mRNAs. At a certain point in embryogenesis, maternal mRNAs are degraded and the zygotic genome becomes active as manifested by an activation of transcription 5, 6, thus developmental control is passed on from maternal components to that of zygotic ones (Fig. 1B). As it turned to be, Neyfakh's ‘morphogenetic function of the nucleus’ consists of the zygotic genome activation (ZGA, i.e., expression of the genome of the embryo proper) and is a crucial part of the mid‐blastula transition (MBT), which also includes the asynchronization of the cell cycle 7 and acquisition of cell motility 8, 9. In fish and frogs, the occurrence of MBT was shown to be dependent on the nuclear to cytoplasmic ratio which increases with each cell division, causing a titration of maternal suppressors which, at a critical point, allows the activation of zygotic transcription 8, 10, 11. In parallel, some studies in insects and mammals led to the development of the maternal‐to‐zygotic transition (MZT) concept, which is equivalent to that of MBT in fish and amphibians. Later on, the concept of MZT was extended to other changes at the molecular as well as cellular level. These include the clearance of maternal mRNAs 12, 13, 14, 15, 16, 17, 18 and major epigenetic rearrangements 19, 20, 21. The consequence of these events culminates in the initiation of gastrulation, during which cells migrate and form the three germ layers.

Despite an absence of transcriptional input from the embryonic genome up to the point of MBT/MZT, developmental processes dynamically progress throughout early embryogenesis. This is possible thanks to the maternally deposited factors, which include a large pool of maternal mRNAs. In order to ensure progression through different developmental milestones, the activity of maternal mRNAs is confined to a precise time and space. In this Review, we will describe how the maternal mRNAs are spatiotemporally organized in the oocyte and early embryo and how their activity is regulated during development.

## The genesis of maternal mRNAs

Before we discuss the regulation of maternal mRNAs, it is necessary to understand their origin. The process of oogenesis in all model organisms studied includes two meiotic arrests. The first arrest occurs during prophase I, which is also known as the period of oocyte growth. During this phase, the oocyte accumulates maternal components required for subsequent maturation and embryonic development. In some organisms such as *Drosophila*, maternal mRNAs are transcribed by cells supporting the oocyte, called nurse cells 22. These maternal RNAs are subsequently transferred to the oocyte by microtubules, cytoskeletal networks, and cytoplasmic streaming 23, 24, 25. During the period of oocyte growth in vertebrates, maternal RNAs are transcribed by the oocyte proper. From this time onward, the oocyte undergoes dynamic changes which include its final maturation, activation, and the earliest steps of embryonic development following its fertilization. These dynamic changes depend on maternal mRNAs activated according to precise timetable to confer functions required.

To prevent premature activation of developmental program, maternal mRNAs transcribed during the oocyte growth undergo translational repression. Their poly(A) tails are shortened immediately after their export into the cytoplasm. Injecting a tPA RNA with a long poly(A) tail into an immature oocyte causes its deadenylation, suggesting that the RNA‐silencing mechanism of oocyte depends on deadenylation 26. The immature oocytes of *Xenopus* and other species contain translationally dormant mRNAs with short poly(A) tails of about 20–40 nucleotides 27, 28, 29, 30, 31, 32, 33. These are stored for activation at appropriate developmental time points.

## Spatial organization of maternal mRNAs in the oocyte and early embryo

All these studies raised a number of important questions, which need to be answered to understand the role of maternal RNAs in more detail. Where do translationally silent maternal mRNAs reside in the egg? What keeps them sequestered from the pervasive translation or degradation machinery in the cytoplasm? And finally, what mechanism ensures precise spatial and temporal activation of maternal mRNAs?

Many maternal mRNAs are spatially localized in the oocyte and early embryos. In *Drosophila*, more than 70% of the early embryonic transcriptome were found to be subcellularly localized 34. Localization of transcripts generally serves to confine their activity and function to the appropriate time and space. In parallel, it also facilitates interactions with important protein regulators. In *Drosophila*, localization of maternal mRNAs in the oocyte is known to be crucial to establish the first embryonic axis 35, while in germ cells, localization of *oskar* and *nanos* mRNAs to the posterior of the germ cell progenitor is necessary to select whether cell will become an oocyte or a nurse cell. As another example, the zebrafish *mos* mRNA is localized to the animal pole, where Zorba protein, required for its translational activation during oocyte maturation, is present 36. During development, differential localization of transcripts into subcellular or subembryonic domains is especially relevant in order to confer asymmetry, which will in turn define embryonic patterning. In early *Xenopus* oocyte, *xdazl* and *nanos1* are localized to the vegetal cortex; in later stage oocytes, vg1 and vegt are transported to vegetal cortex and subsequently inherited during cell cleavage by vegetal blastomeres. Similarly, many maternal mRNAs are localized in zebrafish oocytes. These include the homologs of previously mentioned *Xenopus* mRNAs such as dazl and dvr1 37, 38.

The localization of maternal mRNAs in the fertilized egg and early embryo is driven by several different mechanisms 39, 40. On fertilization in *Xenopus*, the maternal transcripts of xCr1, a coreceptor of the Nodal signaling, are translationally activated in selected cells while repressed in others. This results in the accumulation of xCR1 specifically in the animal and marginal cells and its absence in vegetal cells 41. Localization of mRNAs in the early embryo can occur through cell division. In the early mouse embryo, the cell fate decision between cells of the inner cell mass (ICM) and trophoectoderm occurs due to localization of *Cdx2* mRNA at the apical side of blastomeres at the 8‐ to 16‐cell stage and inherited by daughter cells upon division. Maternally inherited *magoh* transcripts encode a core component of the splicing‐dependent multiprotein exon junction complex deposited at the pre‐mRNA splice junctions. This complex includes several other maternal components (Rbm8A, Casc3, Eif4a3). As a component of exon junction complex, Magoh may influence downstream processes, including nuclear mRNA export, subcellular mRNA localization, translation efficiency, nonsense‐mediated mRNA decay and through its interaction with Pym1 inhibits apoptosis by regulating splicing of BCL2L1/Bcl‐X as well as some other apoptotic factors 42, 43, 44. Magoh is known for its role in germ plasm assembly and germline development in *Drosophila* and zebrafish 38. It is noteworthy that despite its uniform distribution in the 1‐ and 2‐cell stage zebrafish embryo and during late embryogenesis, these transcripts become transiently enriched in the central four blastomeres during 8–16 cell stage 45. This is of interest since these blastomeres contribute to the ectoderm giving rise to the neural tube and epidermis 46, 47. The functional implications of this phenomenon remain to be understood. All we know for now is that a deficiency of one of the members of the exon junction complex, EIF4A3, have been linked to craniofacial abnormalities with learning and language disabilities in human suggestive of defects in neural crest derivatives. In support of this role of EIF4A3, the zebrafish morphants show extensive apoptosis early on and craniofacial phenotype later 48. It remains to be seen how specific is the morphant phenotype and whether an accumulation of *magoh* transcripts in blastomeres giving rise to ectodermal derivatives have any connection to physiological apoptosis in the developing neural tube 49.

In some cases, certain localization signals are known to be crucial for mRNA localization into appropriate subcellular or subembryonic domains. The evolutionary conserved Nodal signaling acts as a determinant of dorsal cell fate and body axis later on. Squint is one of the Nodal ligands in zebrafish. Along with the *Drosophila oskar*,* Xenopus vegt*, the zebrafish *squint* belongs to a group of the so‐called ‘coding and non‐coding’ RNAs (cncRNAs) acting as key developmental regulators in plants and animals 50. At the 4‐cell stage, the maternal mRNA of *squint*, a ligand of the Nodal signaling, was found to be localized to two blastomeres giving rise to dorsal cell fates 51. This localization is driven by a structural motif in the form of a hairpin loop at the 3′UTR of the *squint* transcript 52. This motif is bound by the Ybx1 protein which prevents premature translation of *squint* 53. Of note, prior to the 16‐cell stage, *squint* is translationally repressed and is not polyadenylated. It undergoes cytoplasmic polyadenylation from the 16‐cell stage, consistent with a cohort of maternal mRNAs previously described by our group 53, 54, 55, 56. It is still unclear how the *squint* mRNA is transported to the prospective dorsal blastomeres, although its localization depends on microtubules 51. It is likely that *squint* transport follows a predominant mode of active translocation mediated by the cytoskeletal network of the cell. Interestingly, a similar structure motif was found in other components of the Nodal signaling pathway, which suggests common translational regulation of factors within the same pathway 57.

**Figure 2:**
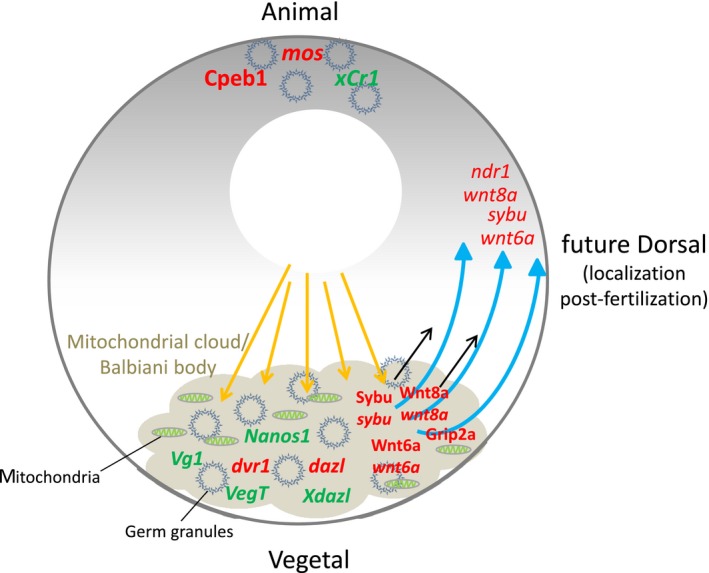
Distribution of key maternal factors in the oocyte. Studies in different organisms have shown that maternal mRNAs are organized in cytoplasmic granules together with several regulatory proteins responsible for their post‐transcriptional processing and thus translational regulation. In fish and amphibians, a large structure known as mitochondrial cloud or Balbiani body is present at the vegetal pole of the oocyte. This structure consists of a large accumulation of mitochondria and cytoplasmic granules (specifically termed germ granules) containing silenced mRNAs. The mitochondrial cloud serves as a vehicle for transporting and localizing maternal factors to the vegetal cortex during oogenesis by means of microtubule network and motor proteins (yellow arrows). At egg activation and fertilization, Sybu and Wnt8 are translocated to the future dorsal axis through microtubule‐mediated transport (blue arrows).

Microtubule‐based transport is known to be involved in localization of dorsal determinants in oocytes and early embryos of amphibians and fishes. In the zebrafish, the mRNA and protein of the maternal factors Syntabulin (Sybu) and Wnt8a are initially localized at the vegetal pole of the oocyte (Fig. 2). On egg activation and fertilization, they are translocated toward the future embryonic dorsal, where they activate the canonical Wnt signaling and induce the body axis 58, 59, 60. This transport was shown to be dependent on arrays of microtubules, which upon egg activation is organized at the vegetal pole of the egg in direction of the future embryonic dorsal side 61. Grip2a, another maternal factor with similar dynamics to that of Sybu and Wnt8a, is responsible for the organization of microtubules at the vegetal pole of the activated egg, which represents the initial step in breaking the asymmetry before the long‐range transport of these factors to the dorsal side 62. The failure of microtubule rearrangement in *grip2a* mutants results in the failure of *wnt8a* mRNA translocation to the future embryonic dorsal and hence axis induction defects. Surprisingly, a more recently performed loss of function analysis of *wnt8a* by Hino and colleagues revealed that maternal Wnt8a is likely to be dispensable for the initial dorsal axis determination 63; nevertheless, it cooperates with zygotically expressed Wnt8a in ventrolateral and posterior tissue formation. The same study also identified Wnt6a as an alternative candidate for dorsal determinant which showed similar expression pattern and localization as *wnt8a*. Among other maternal dorsal determinants located initially at the vegetal pole is Vrtn, whose activity could be suppressed by the putative vegetally localized antagonists. Vrtn binds the *bmp2b* regulatory sequence and represses its zygotic transcription 64.

## Storage of maternal mRNAs in cytoplasmic granules

Transported mRNAs are often present in RNP complexes with RNA‐binding proteins, most of which serve to regulate mRNA processing and repress their translation 65. These RNP complexes are actively transported along the cell's cytoskeletal network in a directed fashion to their rightful subcellular localization. Once the complex reaches its destination, and upon appropriate biological cues, the RNP complex is remodeled and translational activation of these mRNAs ensues. These RNP complexes are often organized into membrane‐less structures known as cytoplasmic granules.

In metazoan cells, structures known as cytoplasmic granules host specific molecular processes pertaining to the inhibition of mRNA translation or degradation 66, 67, 68, 69. These subcellular structures lack membrane and contain proteins involved in specific RNA metabolism function. The organization of maternal mRNAs into cytoplasmic granules would reasonably facilitate their translational suppression by common factors residing in the granules; at the same time, it would also facilitate collective transport to different subcellular domains and rapid response to developmental cues as the lack of membrane of these granules would allow turnover rate in the order of seconds 70. These granules are indeed dynamic in nature—their formation depends on the steady‐state supply of mRNAs subjected to the particular molecular process of the specific granule type. Interestingly, the types of granules present in a cell are regulated in a precise spatial and temporal manner during development as different combinations of granule types each of which perform distinct functions are present in different cell types, according to specific developmental stage 71.

One of the most common and well‐characterized types of cytoplasmic granules are the processing bodies (P‐bodies) known as sites of mRNA repression, decapping, and degradation. P‐bodies contain a core set of decapping enzymes—DCP1 and DCP2, and other accessory proteins such as the DEAD‐box RNA helicase (Dhh1). In the *Caenorhabditis elegans* oocyte, a subset of maternal mRNAs is bound to the homolog of Dhh1 (CGH‐1). Binding of mRNA to CGH‐1 causes its stabilization and repression and directs them to a distinct type of RNA granule 72, 73. Unlike P‐bodies, these CGH‐1 granules lack decapping activity and were found to contain only repressed maternal mRNAs and some interactors of CGH‐1 such as the Ataxin2 and PABP homologs. These translational regulators are also found in stress granules—another type of granules known to sequester and transiently suppress the translation of mRNAs during stress 72. Therefore, in the *C. elegans* oocyte, maternal mRNA association with CGH‐1 and translational regulators nucleates the formation of localized cytoplasmic foci in the form of cytoplasmic granule where translationally repressed maternal mRNAs are stabilized and sequestered.

During oogenesis in *Drosophila*, different types of germ granules exist at different stages and subcellular locations. These granules differ by mRNA composition. However, a single type of granule may differ in mRNA composition at different developmental stages. The germ granules are typically up to 500 nm large and contain core germ plasm proteins Oskar, Vasa, and Tudor as well as other proteins involved in RNA processing 69, 74. They are also sites of active translation containing large numbers of polysomes 75. Sponge bodies are a type of granules found in the nurse cells and oocyte. It is identified by the presence of Exuperantia which is required for the localization of *bicoid* RNA to the anterior pole of the oocyte 25. Sponge bodies exhibit dynamic movements between the nurse cells and oocyte and are known to contain different compositions of mRNAs in different subcellular locations, therefore suggesting dynamic exchange of their contents which is coupled to their transport 76. The polar granule is another type of granule found in the late‐stage oocytes. These granules contain materials transported from the nurse cells to the posterior part of the oocyte. Similar to sponge bodies and P‐granules, the polar granules also contain the decapping enzyme Dcp1 and DEAD‐box helicase Me31B 77, suggesting that these structures are closely related. In addition, they also contain Aubergine and Piwi involved in the piRNA pathway 78, 79. Piwi proteins and piRNAs are thought to protect the germ cells from the mutagenic activity of retrotransposons 80.

In the early *Drosophila* embryo, P‐bodies are sites for translational control of maternal mRNAs required to establish the embryonic axis. By electron microscopy, P‐bodies are identified as electron‐dense structures of about 60–80 nm in diameter‐lacking ribosomes and organized into distinct core and outer region 81. The core contains the *Drosophila* homolog of the DEAD‐box RNA Helicase Dhh1, Me31B. It contains repressed mRNAs and, therefore, may represent the site of translational repression. The outer region contains translational regulator proteins such as Sqd and Orb which are required for translational activation. Active mRNAs are anchored to the outer regions and translated there. Super‐resolution microscopy revealed that mRNAs within the granules form structured, homotypic clusters of single mRNA species localized in a defined position, whereas proteins are evenly distributed throughout the granule 82. Interestingly, the distribution of mRNA clusters within the granule itself does not seem to correlate with their translational activity or their protection from degradation. The homotypic organization of mRNAs into different subcompartments of the granule may allow storage and quick retrieval of specific mRNAs when needed. Such organization may also facilitate localized interactions between regulatory proteins and their target mRNA by providing high local concentrations of the target as well as the protein itself.

In mouse, P‐bodies are found in early‐stage oocytes arrested at prophase I, where they reside in perinuclear foci as well as at the cell cortex 83. The P‐bodies disappear during the period of oocyte growth, and some of their components including cytoplasmic polyadenylation element (CPE)‐binding protein (CPEB) and DDX6 become localized to the subcortical aggregates (SCA), which contain maternal mRNAs. In contrast to the typical P‐bodies, the SCA lack the decapping enzyme DCP1 implicated in mRNA degradation 84. This may reflect the function of SCA in maternal mRNA storage and repression but not degradation. This is similar to the CGH‐1 granules of *C. elegans* 72. On oocyte maturation, the SCA disappear, presumably releasing associated maternal mRNAs from repression.

In *Xenopus* oocytes, maternal mRNAs are localized and translationally repressed in the mitochondrial cloud—an electron dense structure containing germinal granules and associated with dense array of mitochondria (Fig. 2). This structure can be found in stage I oocytes at the future vegetal cortex 85, 86. Within the mitochondrial cloud, silenced mRNAs are organized within or around the germinal granules which aggregate at the vegetal pole. The mitochondrial cloud delivers germinal granules and localizes maternal mRNAs to the vegetal cortex by the messenger transport organizer (METRO) pathway during stage I and II of oogenesis. This mechanism works through the entrapment of germ plasm mRNAs by the dense ER network of the mitochondrial cloud and subsequent expansion of the cloud toward the vegetal cortex 87, 88.

A structure termed the Balbiani body analogous to the mitochondrial cloud could also be found in zebrafish 89. This structure resembles the mitochondrial cloud in that it associates with mitochondria as well as germ granules and their associated maternal mRNAs (Fig. 2). The Balbiani body is also known to be involved in transporting and localizing maternal mRNAs to the oocyte vegetal cortex 90, 91. The formation of the Balbiani body is linked to orientation of the chromosomal bouquet, a specific organization assumed by the chromosomes in the nucleus at the onset of meiosis, in a microtubule‐directed process 92. Subsequent to its formation, the Balbiani body associates with the oocyte cortex and releases its mRNPs containing most of the dorsal determinants and germ plasm components, including *sybu* and *wnt8* discussed in previous section 38, 91, 93. The establishment of the Balbiani body at the vegetal pole, therefore, determines the polarity of the oocyte, which is in turn necessary to establish the embryonic axis and specify the germ line 61, 94, 95. To date, very few studies were performed on cytoplasmic granules in zebrafish embryogenesis or for that matter during the embryogenesis of vertebrates. However, it is known that the activation of zygotic transcription results in the formation of distinct types of nuclear granules including those involved in the processing of histone mRNAs and assembly of splicing machinery 96. Given the nature of cytoplasmic bodies in other systems known to be dependent on the presence of mRNA itself, it is plausible that the activation of zygotic transcription represents a trigger for the formation of such granules.

## The timely activation of maternal mRNAs at developmental milestones

**Figure 3:**
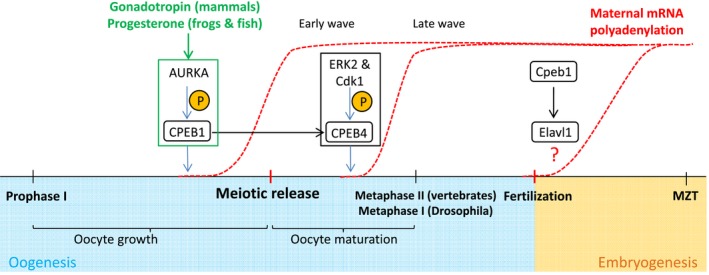
Different waves of maternal mRNA activation throughout oogenesis and early embryogenesis mediated by cytoplasmic polyadenylation. Maternal mRNAs are synthesized throughout the period of oocyte growth and stored in a dormant state. The first wave of activation is stimulated by GH or progesterone, resulting in phosphorylation of CPEB1 by Aurora kinase and the cytoplasmic polyadenylation of several maternal factors required for oocyte maturation. Another late wave is in turn mediated by CPEB4 whose translation was activated during the early wave and is phosphorylated by ERK2 and Cdk1 kinases. Following fertilization, another wave of cytoplasmic polyadenylation occurs on thousands of maternal transcripts. This later wave is required for the embryo to undergo a proper process of MZT. However, the exact molecular mechanism regulating this third wave of cytoplasmic polyadenylation has not yet been worked out.

Up to the point of ZGA, the dynamic processes of oogenesis and embryogenesis rely solely on maternal mRNAs. The absence of transcriptional input from the zygotic genome necessitates regulation of maternal RNAs at the post‐transcriptional level in order to activate different subsets of mRNAs with precise developmental timing. Over the last two decades, several studies have identified translational control by cytoplasmic polyadenylation as one of the mechanisms to regulate the timely activation of maternal mRNAs during the development prior to MZT 28, 33, 97, 98, 99, 100, 101, 102, 103, 104, 105. In the mouse, *Xenopus*, and zebrafish, maternal mRNAs encoding key drivers of oogenesis and early embryonic development are deadenylated right after their synthesis during oocyte growth and stored to be reactivated at appropriate developmental milestones (Fig. 3). These deposited maternal mRNAs have a short poly(A) tail and are translationally inactive. A portion of the stored maternal mRNAs becomes dramatically adenylated at specific stages of oogenesis as well as upon fertilization, which correlates well with their recruitment into polysomes and translation initiation 29, 30, 56, 106, 107, 108. In *Xenopus* oocytes, the poly(A) tail could increase by as much as twofold or more, resulting in a corresponding increase of translation efficiency 29, 30, 108.

The initiation of oocyte maturation represents the first point of activation of stored maternal mRNAs. In most vertebrates, upon ovulation, meiosis is reinitiated and arrested a second time in metaphase II 109, whereas in *Drosophila*, meiotic arrest takes place in metaphase I 110. The point of meiotic release from prophase I is known as oocyte maturation which occurs upon stimulation by hormones—gonadotropin in mouse 73 and progesterone in *Xenopus* and zebrafish 111, 112 or exposure to a particular signal released by sperm in *C. elegans* 113, 114. During its maturation, the oocyte undergoes two consecutive M‐phases without an intervening S‐phase. As a result of this maturation, the oocyte can be fertilized, upon which the completion of meiosis ensues and embryonic development initiates. One of the earliest transcripts to be translationally activated upon oocyte maturation is c‐Mos kinase 115. The activity of this kinase is crucial to initiate a subsequent series of cytoplasmic polyadenylation and translation of other mRNAs’ encoding factors essential for oocyte maturation such as Cyclin B1 116, 117, 118, 119. Loss of function of these factors, overexpression, or prevention of their translational activation was shown to prevent oocyte maturation, indicating that their timely activation is essential 119, 120. In the zebrafish and mouse oocytes, *cyclin B1* mRNAs are repressed and stored in cytoplasmic RNA granules which disassemble during oocyte maturation. The formation of these granules is required for the translational repression, while their disassembly coincides with translational activation of *cyclin B1* mRNAs. Disruption of the granules results in premature translation of Cyclin B1, which negatively affects oocyte maturation 121.

The second event of maternal mRNA activation occurs during embryonic development, which in some species is triggered by fertilization. In zebrafish, a large cohort of maternal mRNA was found to undergo cytoplasmic polyadenylation prior to the MBT 54. This process was shown to correlate with the increase in mRNA association with polysomes. It is essential for developmental progression past MBT/MZT 56.

## The molecular machinery of cytoplasmic polyadenylation

Polyadenylation of eukaryotic mRNAs is an inherent part of gene expression. It involves the addition of multiple adenine bases to the 3′ end of the mature mRNA. Polyadenylation of mRNA is tightly linked to its stability and translatability. All mRNAs undergo nuclear polyadenylation following their transcription and maturation 122, 123. This process is dictated by a hexamer motif at the 3′UTR of the mRNA known as the nuclear polyadenylation sequence (AAUAAA) 30. While some mRNAs are translated upon translocation to the cytoplasm, others are deadenylated upon reaching cytoplasm and stored to be translated at a later stage 26. The latter group of mRNA possesses an additional signal in their 3′UTR known as the CPE, a sequence signal at the 3′‐UTR of mRNA molecules, which determines whether the mRNA should undergo such translational regulation 124, 125. This mode of translational regulation is known as cytoplasmic polyadenylation.

The molecular machinery of cytoplasmic polyadenylation is probably best characterized within the context of oocyte maturation and early embryogenesis in *Xenopus* 106, 107, 126. The CPEB represents a family of RNA‐binding proteins. They bind CPE to regulate translation by nucleating the formation of cytoplasmic polyadenylation complex as well as translational enhancing and repressing complexes 124, 127, 128. All CPEB proteins contain a highly conserved C‐terminal, which consists of two RNA recognition modules responsible for binding target RNAs and a less conserved N‐terminal domain. The latter contains phosphorylation domains and protein–protein interaction domains presumed to be functionally important 129, 130, 131, 132. In the vertebrates, the two classes of CPEBs are represented by CPEB1 and CPEB2‐4 groups 127. The latter group is found to be expressed abundantly in the central nervous system. It is known to function in synaptic plasticity and long‐term memory formation 133, 134, 135. CPEB1 and CPEB4 play a role in oogenesis and are activated through different mechanisms. To be activated, CPEB1 is phosphorylated by the Aurora kinase A (Aurka), whereas CPEB4 by the ERK2 and Cdk1 kinases 136. CPEBs perform a dual role in translational regulation: in their native unphosphorylated form, they repress cap‐dependent translation; in their phosphorylated active form, they activate the translation of mRNAs through promoting cytoplasmic polyadenylation. CPEB1 recognizes the CPE at the 3′UTR of the mRNA molecule. The recognition of target mRNAs by CPEBs occurs in the nucleus. These mRNAs by default have long poly(A) tails. When the mRNAs are exported to the cytoplasm, CPEB nucleates the formation of a repressive complex, which includes the Gld2 poly(A) polymerase and PARN poly(A) ribonuclease. As PARN is more active, the concurrent activity of these two proteins results in the net shortening of poly(A) tail. On appropriate biological stimuli, PARN is expelled from the complex, resulting in elongation of the poly(A) tail due to Gld2 activity 128. The long poly(A) tail is subsequently recognized by the poly(A)‐binding protein (PABP), which recruits the translation initiation complex and initiates mRNA translation 137.

## Cytoplasmic polyadenylation mechanism in oogenesis and early embryonic development

In the *Xenopus* oocyte, a heterodimeric complex of CDC2 and Cyclin B, known as the maturation‐promoting factor (MPF) induces the initial entry into metaphase I from prophase I 116. In oocytes arrested at prophase I, this complex is initially inactive and requires CDC25 for its activation. Progesterone stimulation results in the phosphorylation of Cpeb1 by Aurka (Fig. 3), resulting in the earliest wave of cytoplasmic polyadenylation of mRNAs encoding the components required for activation as well as the assembly of the MPF 125. Importantly, this early wave of polyadenylation also results in the translation of Cpeb4 which is responsible for a second late wave of polyadenylation during the second meiotic division (Fig. 3). Cpeb4 is activated through a distinct mechanism. ERK2 and Cdk1 phosphorylate Cpeb4 at its N‐terminal domain which contains an intrinsically disordered region. This hyperphosphorylation maintains Cpeb4 in a monomeric active state required for cytoplasmic polyadenylation 136. Otherwise, Cpeb4 forms liquid‐like droplets by means of its N‐terminal region, sequestering mRNAs and suppressing their translation.

Besides Cpeb1 which is implicated in oocyte maturation, an additional small CPEB‐like RNA‐binding protein (ElrA) is present at the earliest steps of embryonic development in *Xenopus*. ElrA recognizes the embryonic‐type CPEs present in maternal mRNAs regulated during embryogenesis 106, 126, 138. It is a member of the ELAV family of RNA‐binding proteins implicated in regulating mRNA stability, translation, and transport in *Drosophila* and mammalian neurons 139. The zebrafish homolog of ElrA, *elavl1*, was shown to be under translational regulation through cytoplasmic polyadenylation during pre‐MBT stages 140 Moreover, the translation of *elavl1* appeared to be regulated by Zorba, the zebrafish homolog of CPEB1 141.

Previous research from our group revealed that at least 3000 of maternal transcripts undergo progressive increase in poly(A) tail prior to MBT 54. Subsequently, Subtelny and colleagues 108 showed the correlation between poly(A) tail elongation and translation efficiency of maternal mRNAs in several organisms, including the zebrafish. A unifying observation from these studies is that embryonic cytoplasmic polyadenylation seems to occur almost exclusively during the period of transcriptional quiescence 54, 108. Inhibition of this process by genetic mutations or chemical treatment of embryos with 3′ deoxyadenosine (3′dA) has been shown to result in the failure of passing through the MBT milestone in zebrafish 54, 56 and MZT in other organisms 97, 100. The transcripts of at least three different CPEBs—*cpeb1/zorba, cpeb4a*, and *cpeb4b,* were maternally supplied 54, 56. In addition, *elavl1* is also present at this stage 138. Each of these factors show distinct expression dynamics 54, 56, 138, which suggests their sequential roles in regulating cytoplasmic polyadenylation and translational activation of maternal mRNAs in a timely manner. *Zorba* transcripts are initially present at high level from egg stage and are gradually degraded as development proceeds, in agreement with its known role in oocyte maturation in *Xenopus*. The degradation of *zorba* coincided with the gradual increase in cytoplasmic polyadenylation of *cpeb4a, cpeb4b*, and *elavl1* (in agreement with 140. While the level of *elavl1* continues to increase post‐MBT, *cpeb4* levels immediately fall following its peak expression at MBT. Moreover, in agreement with their known roles in later developmental processes 133, 134, 135, *cpeb2* and *cpeb3* were undetectable during the pre‐MBT stages, with *cpeb2* starting to be expressed only after MBT 54, 56. The sequential expression patterns of the different CPEBs coincide with the different developmental milestones and thus suggest distinct targets and function at different stages of development.

## Mechanistic insights from studies of cytoplasmic polyadenylation and CPEB beyond embryogenesis

Besides its role in oogenesis and embryogenesis pre‐MBT/MZT, cytoplasmic polyadenylation also plays an important role in later stages of life. The best characterized examples of cytoplasmic polyadenylation in somatic cells are known to occur in the central nervous system 133, 134, 135, in synaptic junctions as part of a mechanism for the establishment of long‐term memory. It is known that memory formation is dependent on protein synthesis for the duration of the training stimuli 142, 143, 144. CPEB plays a role in synaptic plasticity and neuronal morphology and hence determines learning and long‐term memory 145, 146, 147, 148. In the neurons, CPEB is localized in RNP granules distributed along the dendritic arbor and localized in the vicinity of synapses 128, 149. The CPEB regulation of localized translation in the synapses has been observed in the sea slug *Aplysia* 150. Several key evidences support this idea as disruption of CPEB function results in abnormalities in various aspects of neuronal development and function 133, 151, 152.

Although the role of CPEB characterized so far is predominantly within the cytoplasmic domain, CPEBs also have a prominent role in the nucleus. CPEB1 proteins were observed to be shuttling between the cytoplasm and nucleus in cultured cells as well as in *Xenopus* embryos 153, 154, 155. It is well established that post‐transcriptional mRNA regulation usually starts even before cytoplasmic import, where RNA‐binding proteins regulating translation shuttle between nucleus and cytoplasm. They bind nuclear mRNAs and regulation of translation can occur once they are exported to the cytoplasm. In this sense, the behavior of CPEB1 is in agreement with this concept. In the nucleus of HeLa cells, CPEB1 is associated with nucleolar bodies containing the nuclear export protein Crm1. In the *Xenopus* oocytes, CPEB1 associates with lampbrush chromosomes. Interestingly, this association is RNAse‐sensitive, showing that CPEB1 binds to nascent‐transcribed mRNAs known to associate with proteins involved in nuclear RNA processing 154. These observations suggest that CPEB1 may play a role in processing of RNAs and translational control. However, despite this knowledge, the exact role of CPEB1 in the nucleus was not known until relatively recently. Bava and colleagues 156 observed that CPEB1 in the nucleus colocalizes with splicing factors. Here, CPEB1 promotes the formation of alternative 3′UTRs of mRNAs: its binding to target mRNAs promotes the usage of a more proximally located alternative polyadenylation site (PAS), resulting in a shorter 3′UTR. In this scenario, CPEB1 recruits the cleavage and polyadenylation specificity factor to a more upstream and weaker PAS. Under normal circumstances, the stronger downstream PAS would be used, thus resulting in a transcript with longer 3′UTR. It is thought that having a shorter 3′UTR would subject the transcript to less translational regulation, as it would contain less regulatory elements. Hence, the presence of CPEB1 would result in more efficient translation of target transcripts. An example of this mechanism could be observed in the liver, where CPEB1 mediates the processing of the 3′UTRs of VEGF and CPEB4 mRNAs. This results in a shorter isoform of both mRNA species lacking translational repression and mRNA destabilizing elements, which results in the overall increase in their translation 157. The consequent increase in CPEB4, in turn, leads to the increase in cytoplasmic polyadenylation of the VEGF mRNA, which stimulates VEGF translation further. In the human liver, CPEB1 and CPEB4 are expressed uniformly at low levels. In patients with cirrhotic liver, however, their expression is strongly increased particularly in hepatocytes at the regenerative foci 157. In addition, CPEBs and VEGF expression is also elevated in the endothelium of new vessels generated in fibrotic areas, which is thought to stimulate endothelial cell recruitment and proliferation necessary for angiogenesis. The loss of function of CPEBs was found to limit the pathologic progression of VEGF‐mediated angiogenesis without compromising physiological function of VEGF, which could thus be explored further as a potential therapeutic target to prevent disease progression.

## Extending the morphogenetic function

Besides cytoplasmic polyadenylation, many other means of translational regulation which are not covered in this Review have been characterized in oogenesis and embryogenesis 158. In case of the latter, most of the forms known are suppressive in nature, the end result of which is often the instability of the transcript and its degradation 158, 159, 160. One mechanism which recently came to light is codon optimality 161 which has been demonstrated to regulate maternal mRNA clearance in the zebrafish 162, 163. With the advent of large‐scale analysis of genomes and transcriptomes brought about by high‐throughput next generation sequencing (NGS) technologies, particularly those which enables the profiling of global translation 164, 165, it is reasonable to expect that more and more of such mechanisms will come to light in the near future, which will enable us to gain a more comprehensive understanding of maternal RNA biology. By extension, the routine comparison of large numbers of maternal and zygotic transcripts at the genomic level has driven rapid progress in understanding of the molecular mechanisms of the MBT which had been discovered by Neyfakh in 1950s. Hence, his papers were rediscovered by a new generation of developmental biologists 54, 56, 166. And yet, there was a second part of the MBT story that for now remains hidden in the libraries. One intriguing continuation of the studies on ‘morphogenetic’ function of nuclei (defined as the ability to support embryonic development) has been the ‘vitogenetic’ function (defined as the ability of irradiated embryos to survive without developmental progression 2. In continuation of the ‘vitogenetic’ function story, it was found that the microinjection of a fraction of small nuclear RNA (snRNA) known for their role in pre‐mRNA splicing 167, but not any other RNA extracted during mid‐gastrula embryos into irradiated 1–2 cell stage loach embryos led to a significant increase of zygotic transcription as well as noticeable extension of the period of their survival. In this case, the zygotic transcription has been detected by cultivating the isolated blastoderms at specific stages (7 hpf, note that at optimal temperature, the loach develops approximately twice slower compared to zebrafish) with radioactively labeled precursors of DNA, RNA, and protein for 1 h and measuring radioactivity of the trichloroacetic acid filter precipitate in the liquid scintillation counter. The relative increase of transcription as defined by incorporation of tritiated uridine was higher comparing to replication and protein synthesis as defined by incorporation of labeled thymidine and leucine, correspondingly 168, 169, 170. In other words, an excess of snRNA seems to cause a prolongation of the ‘vitogenetic’ function 171. What could be the reason for this?

It has been shown that, upon fertilization, the *Xenopus* egg contains a certain amount of snRNA U1, sufficient for 4000–8000 nuclei, that is, roughly corresponding to the number of cells at the MBT. In addition, when zygotic transcription is activated at the twelfth cleavage (4000‐cell stage), RNA polymerase II generates mainly snRNAs. From the twelfth cleavage to gastrulation, U1 RNA increases sevenfold in parallel with increase of cell number. This level of snRNA transcription is much above that present in somatic cells. This suggests much higher U1 transcription or a greater number of active U1 genes in the embryo, suggesting the critical developmental role of snRNA transcripts. Interestingly, microinjection of U1 snRNAs into the cytoplasm of a mature *Xenopus* oocyte triggers the transport of snRNA‐binding proteins into the nucleus (germinal vesicle, GV) 172. In the sea urchin, the maternal U1 RNA and U1‐specific ribonucleoproteins (snRNP) reside in the GV. On oocyte maturation, they relocate to the cytoplasm where they are maintained during early cleavage and U1 RNA appears in the nuclei of micromeres at the 16‐cell stage. In contrast, the zygotic U1 transcripts are confined to nuclei, while the maternal ones remain cytoplasmic. Being released to the cytoplasm at oocyte maturation, these RNPs (or RNAs) may have been structurally altered 173. Intriguingly, the snRNA themselves are a subject of cytoplasmic polyadenylation 174, 175. It remains to be seen whether the 1988 experimentally induced elongation of the ‘vitogenetic’ function could be due to a strictly limited mass of maternal snRNA or its structural modification involving cytoplasmic polyadenylation. Whatever the answer, it seems that there are multiple fractions of maternal RNAs whose activation during development involves cytoplasmic polyadenylation.

The understanding of translational regulation mechanism extends beyond early embryonic development and could be extrapolated to different biological aspects. With the development of high‐throughput sequencing techniques, it is now possible to move beyond single‐gene studies and obtain a global view of translation which has often been limited by technical challenges associated with protein analyses. Incorporation of the increasing number of such datasets into the established knowledge of the principles of early embryonic development would be the next step to establish a more comprehensive understanding of translational regulation of maternal mRNAs. One caveat, however, is that this methodology is relatively easy to use for the analysis of transcripts within the temporal aspect, whereas it remained more challenging to use this methodology to resolve developmental processes in space, taking place in 3D environment of oocytes and embryos. This situation changes for the better with the introduction of single‐cell transcriptome analysis. In the zebrafish field, the feasibility of this technique have recently been demonstrated to construct the spatial map of the early zebrafish gastrula based on the expression profiles of individual cells 176 and for lineage tracing of individual cells in the early zebrafish embryo in combination with CRISPR/Cas9 gene editing 177. It is foreseeable in the future that the application of single‐cell‐based techniques, in combination with whole embryo analysis, will open up vast possibilities to study and unravel novel aspects of the regulation of maternal mRNAs in the spatial context.

## Funding

CLW is supported by the EU FP7 Grant Fishmed GA No. 316125, Polish National Science Center (NCN) OPUS grant No. 2014/13/B/NZ2/03863, and the FNP First Team grant GA No. First TEAM/2016‐1/8. VK is supported by Polish National Science Center (NCN) OPUS grant No. 2016/21/B/NZ3/00354.
